# Rapid Differential Detection of Wild-Type Classical Swine Fever Virus and Hog Cholera Lapinized Virus Vaccines by TaqMan MGB-Based Dual One-Step Real-Time RT-PCR

**DOI:** 10.3390/vetsci11070289

**Published:** 2024-06-28

**Authors:** Yongzhe Zhang, Meiqi Wang, Yajuan Sun, Xingyu Xiao, Songsong Wang, Peng Li, Yansong Liu, Hongri Zhao, Yan Meng, Rui Yin

**Affiliations:** 1 College of Biological and Pharmaceutical Engineering, Jilin Agricultural Science and Technology University, Jilin 132101, China; 2 Department of Neurology, China-Japan Union Hospital of Jilin University, Changchun 130033, China; 3 Research and Development Center, Sairuisi Biotechnology (Jilin) Co., Ltd., Changchun 130102, China

**Keywords:** CSFV, HCLV, TaqMan-MGB probes

## Abstract

**Simple Summary:**

Classical swine fever is a highly contagious disease that severely affects the swine industry worldwide. The clinical symptoms of infected pigs are unusually complex, making it difficult to distinguish between wild-type and vaccinated pigs. In this study, a dual TaqMan-MGB RT-qPCR method for the identification of wild-type CSFV and attenuated vaccine strains was established. This newly developed method could specifically detect CSFV and HCLV with no cross-reactivity with other swine pathogens. The detection limit for the *NS3* gene of CSFV and HCLV was 1.67 × 10^1^ copies/μL, respectively. For precision testing, the repeatability and reproducibility of the relative standard deviation was less than 2%. This method was successfully used for rapid detection of 193 real samples. This dual TaqMan-MGB RT-qPCR technology meets the needs for early and rapid detection of CSFV, and can provide an efficient and rapid detection method for the investigation and analysis of CSFV field strain infections and vaccine immune status, thereby laying the foundation for better prevention and control of the occurrence and spread of CSF.

**Abstract:**

To establish a rapid real-time RT-PCR method for differentiating wild-type classical swine fever virus (CSFV) strains from vaccine strains (HCLV), we designed a universal primer targeting the *NS3* gene to detect wild-type CSFV strains and vaccine strains simultaneously, and two TaqMan-MGB probes were designed to differentiate between wild-type and vaccine strains. After optimizing the RT-qPCR conditions, a rapid dual TaqMan-MGB RT-qPCR method for the detection and identification of CSFV and HCLV was developed. The results showed that method could specifically detect CSFV and HCLV with no cross-reactivity with other swine pathogens. The analytic sensitivity for the *NS3* gene of CSFV and HCLV were 1.67 × 10^1^ copies/μL, respectively. For precision testing, the repeatability and reproducibility of the test was less than 2%. This method was successfully used for the rapid detection of 193 biological samples collected from CSFV-vaccinated pigs. This fast and accurate detection technology can be used for the detection of CSFV and is suitable for differentiating between wild-type CSFV strains and vaccine strains.

## 1. Introduction

Classical swine fever (CSF) is a highly contagious disease characterized by high fever and hemorrhage [1]. CSF has a severe impact on the swine industry and is listed as an A-class legal infectious disease by the World Organization for Animal Health (WOAH) [2]. CSF is caused by the classical swine fever virus (CSFV), which belongs to the Flaviviridae family and the Pestivirus genus [3]. Pigs of all ages can be infected with CSFV, which has strong infectivity, rapid spread, and causes a high mortality rate [4]. The clinical manifestations of CSF include fever, cough, ataxia, conjunctivitis, diarrhea, stillbirth, and abortion [5,6].

CSFV is an enveloped, positive-sense, single-stranded RNA virus with a genome size of 12.3 kb [7]. Based on the differences in the sequences of E2 gene, 5′ non-coding region (5′ NCR) and the polymerase gene (NS5B), CSFV can be divided into three genetic groups: I, II, and III [8]. Phylogenetic analysis indicated that strain C (vaccine strain) belongs to genetic group I and can elicit immune protection against highly virulent strains and various genetic groups. Epidemiological investigations of CSFV in China have shown that all wild-type CSFV strains belong to genetic group II [9], and the widespread use of the C strain vaccine makes it difficult to distinguish between the wild-type and C strains, even during laboratory testing [10]. Currently, there are reports on the use of real-time RT-PCR to differentiate wild-type CSFV strains from the C strain [11]. The TaqMan MGB probe is a molecular beacon based on the principle of fluorescence resonance energy transfer (FRET). It can detect single-nucleotide mismatches and achieve high-precision detection of gene mutations with a high sensitivity and specificity [12]. Additionally, MGB probes exhibit high stability, allowing for hybridization reactions under high-temperature conditions, thus avoiding the issue of easy degradation encountered by traditional molecular beacons at room temperature [13,14]. To facilitate the rapid identification of classical swine fever virus and to differentiate between wild-type CSFV infections and vaccination with attenuated vaccines, this study established a dual TaqMan-MGB RT-qPCR method for the identification of wild-type CSFV and attenuated vaccine strains.

In this study, a pair of universal primers was designed within the NS3 gene to detect the wild-type CSFV and the vaccine strain simultaneously, and two TaqMan MGB probes were designed to detect and differentiate between the wild-type and C strains. This dual TaqMan-MGB RT-qPCR provides an effective detection method for the rapid and accurate detection of wild-type CSFV and C strains in the swine industry.

## 2. Material and Methods

### 2.1. The Strains Used in This Study

Five reference strains of CSFV (Brescia, Shimen, RUCSFPLUM, Paderborn, SSFV39) and vaccines with a titer of 10^4.7^ TCID_50_/μL–10^5.5^ TCID_50_/μL, were obtained from Sairuisi Biotechnology (Jilin) Co., Ltd., Changchun, China. And other commerce vaccines include classical swine fever virus vaccine (CVCC AV1412), Chinese Swine Fever Rabbit Attenuated Vaccine Strain (HCLV), High-pathogenicity Porcine Reproduction and Respiratory Syndrome vaccine, live (Strain JXA1-R); Porcine Reproduction and Respiratory Syndrome vaccine, live (Strain R9); Porcine Reproduction and Respiratory Syndrome vaccine, live (Strain CH-1R); Porcine Reproduction and Respiratory Syndrome vaccine, inactivated (Strain CH-1a); Swine Epidemic Encephalitis Vaccine, live (Strain SA14-14-2); Porcine Parvovirus vaccine, inactivated (Strain WH-1); Swine influenza virus H1N1 subtype, inactivated vaccine (TJ strain); Porcine pseudorabies vaccine, live vaccine (HB-98 strain); Classical Swine Fever Live Vaccine (Cell-Derived) (CVCC AV1412 Strain); Inactivated porcine circovirus disease type 2 vaccine (SH strain); Porcine transmissible gastroenteritis, porcine epidemic diarrhea, porcine rotavirus (GP5 type) live vaccine (flower virus strain + CV777 strain + NX strain); Foot and mouth disease type O and A bivalent 3B protein epitope deletion vaccine, inactivated (O/rV-1 strain + A/rV-2 strain); Haemophilus parasuis quadrivalent propolis inactivated vaccine (type 4 SD02 strain + type 5 HN02 strain + type 12 GZ01 strain + type 13 JX03 strain); Staphylococcus suis propolis inactivated vaccine (Staphylococcus suis group C BHZZ-L1 strain + Staphylococcus suis type 2 BHZZ-L4 strain); Trivalent inactivated vaccine for virulent colibacillosis in piglets (containing K88, K99, and 987P flagellar antigens); Porcine Mycoplasmal Pneumonia Inactivated Vaccine (Strain J); Triple live vaccine for swine fever, swine erysipelas and pasteurellosis (cell source + G4T10 strain + EO630 strain); Inactivated vaccine against porcine atrophic rhinitis (Bordetella pertussis strain JB5); Porcine infectious pleuropneumonia trivalent inactivated vaccine (serotype 1 9901 strain, serotype 2 XT9904 strain, serotype 7 GZ9903 strain). All these commerce vaccines were preserved at −20 °C in our laboratory. The manufacturers’ information for commercial vaccine strains are in supplementary Table S1.

### 2.2. Main Reagents and Instruments

A viral magnetic bead-based DNA/RNA extraction kit and bacterial magnetic bead-based DNA extraction kit were purchased from Sairuisi Biotechnology (Jilin) Co., Ltd. (Changchun, China). The Hifair^®^ V C58P2 Multiplex One Step RT-qPCR Probe Kit (UDG Plus) was purchased from Yeasen Biotechnology (Shanghai) Co., Ltd., Shanghai, China. The 4S GREEN dye, 2000 DNA Marker, 500 DNA Marker, M-MuLV First Strand cDNA Synthesis Kit, and gel extraction and purification kit were purchased from Sangon Biotechnology Co., Ltd. (Shanghai, China). A classical porcine swine fever real-time fluorescence RT-PCR detection kit was purchased from Beijing Anheal Laboratories Co., Ltd., Beijing, China. A real-time PCR instrument (Gentier 96R) was purchased from Xi’an Tianlong Biotechnology Co., Ltd. (Xi’an, China).

### 2.3. Preparation of Nucleic Acids from Pathogenic Microorganisms

The viral DNA/RNA and the bacterial DNA were extracted using the viral and bacterial magnetic bead-based DNA extraction kits, respectively, and the RNAs of the RNA virus were reverse transcribed using the First-Strand cDNA Synthesis Kit and stored at −20 °C for later use.

### 2.4. Primer and Probe Design

**Figure 1:**
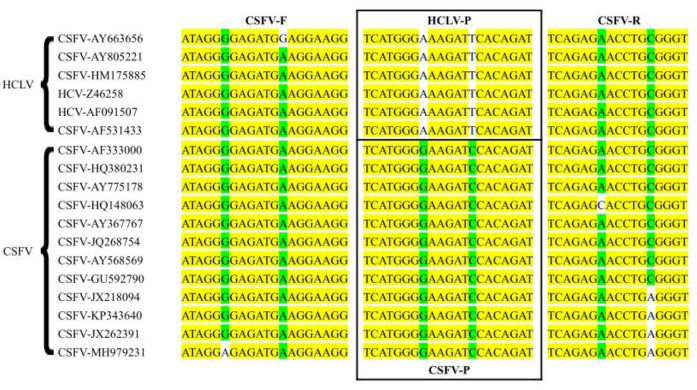
Sequence alignment information for primers and probes. The yellow labeled area represents the conserved region of the genes; the green and white labeled area represents the variant site of the genes.

The full genome sequences of CSFV (GenBank NO: AF333000) and HCLV (GenBank NO: AY805221) were downloaded from GenBank, and multi-sequence alignment analysis was performed using the alignment program in the Vector NTI v11.0 (Thermo Fisher Scientific, Waltham, MA, USA). Primers were designed based on the *NS3* gene site, and two TaqMan-MGB probes were designed based on the SNP sites in the *NS3* gene to differentiate CSFV wild-type strains from the C strain (Figure 1). The specificity of the primers and probes was validated using the BLAST tool (https://blast.ncbi.nlm.nih.gov/Blast.cgi, accessed on 6 September 2023). The primer and probe sequences are listed in Table 1. The primers and probes were synthesized by Sangon Biotechnology Co., Ltd. (Shanghai, China).

### 2.5. Preparation of Standard Positive Plasmids

CSFV and HCLV RNA were amplified using conventional reverse RT-PCR. The target NS3 genes were recovered from the agarose gel, and ligated with the pUC57 and pUCm -T vector, respectively. The ligation mixtures were transformed into *E. coli* DH5α competent cells, and the positive clones were selected; these clones were identified by PCR and sequencing. The correct clones containing NS3 genes were named CSFV-NS3-Plasmid and HCLV-NS3-Plasmid. According to the formula: Y (copies/μL) = (6.02 × 10^23^ copies/mol) × (X ng/μL × 10^−9^)/(DNA length × 660). The concentrations of the plasmids of CSFV-NS3-Plasmid and HCLV-NS3-Plasmid were 3.34 × 10^10^ copies/µL and 5.8 × 10^10^ copies/µL, respectively.

The CSFV-NS3-Plasmid and HCLV-NS3-Plasmid were diluted by a 10-fold serial dilutions, respectively. The two plasmids at the same concentration level were mixed at a ratio of 1:1 (*v/v*), and the concentration of the plasmid mixtures (CSFV-NS3-Plasmid and HCLV-NS3-Plasmid) was 1.67 × 10^10^ copies/µL to 1.67 × 10^0^ copies/µL.

### 2.6. Optimization of Dual TaqMan-MGB RT-qPCR

To obtain an optimal RT-qPCR reaction system, 10^7^ copies/μL, 10^6^ copies/μL, and 10^5^ copies/μL of the plasmid mixtures were used as the template for dual TaqMan-MGB RT-qPCR. The primer concentration was set at 0.2, 0.4, and 0.6 μmol/L (the probes’ concentrations were 0.2 μmol/L), after the optimal primer concentration was determined, the probes’ concentrations (0.1, 0.2, 0.3, and 0.4 μmol/L) were also tested. After the primers’ and probes’ concentrations were determined, the annealing temperature (58 °C, 60 °C, and 62 °C) was optimized.

### 2.7. Establishment of the Standard Curve

To establish the standard curve, 10^3^ copies/µL to 10^7^ copies/µL of the plasmid mixtures were used as the DNA templates and then amplified by dual TaqMan-MGB RT-qPCR method. For the standard curve, each concentration was run in triplicate.

### 2.8. Analytic Specificity

To validate the specificity of the assay for CSFV detection, a dual TaqMan-MGB RT-qPCR was employed for CSFV detection, and HCLV and other pathogens (Section 2.1) were used for cross-reactivity testing. At the same time, to evaluate the specificity of the assay for HCLV detection, dual TaqMan-MGB RT-qPCR was employed for HCLV and CSFV detection, and other pathogens mentioned above were utilized for cross-reactivity testing.

### 2.9. Analytic Sensitivity

To determine the analytic sensitivity of dual TaqMan-MGB RT-qPCR, 10^0^ copies/µL to 10^8^ copies/µL of the plasmid mixture were used as the DNA templates, with ddH_2_O as the negative control. The analytic sensitivity of the dual TaqMan-MGB RT-qPCR was evaluated. Besides that, 10-fold gradient dilution of CSFV and HCLV attenuated vaccines, ranging from 10^5.5^ TCID_50_/tube to 10^−0.5^ TCID_50_/tube. Dual TaqMan-MGB RT-qPCR was conducted to assess the sensitivity of the detection, using ddH_2_O as the negative control.

### 2.10. Inclusiveness of the Test

Five classic epidemic strains of CSFV (Brescia, Shimen, RUCSFPLUM, Paderborn, SSFV39) were chosen for inclusiveness assessment of the test. The strains were diluted to the lowest limit of detection of the test, and then each of these strains was tested 3 times using dual TaqMan-MGB RT-qPCR technology.

### 2.11. Precision of the Test

The reproducibility of the experiment was evaluated using a moderately positive sample consisting of a plasmid mixture at a concentration of 10^3^ copies/µL, a weakly positive sample with a plasmid mixture at a concentration of 10^1^ copies/µL, and a negative control composed of deionized water (ddH_2_O); the same batch of dual TaqMan-MGB RT-qPCR solution was tested three times.

For the reproducibility of the test: this test was performed at two laboratories [including Molecular Diagnostic Science and Technology Innovation Center of Jilin Agricultural Science and Technology University, and Research Laboratory of Sairuisi Biotechnology (Jilin) Co., Ltd.], with two brands of qPCR instruments (Gentier 96R, Tianlong Xian, China and StepOne Plus, Applied Biosystem, Foster City, CA, USA), and two operators and two batches of reagents (batch numbers 20240608 and 20240610) were used for qPCR amplification. Each dilution was repeated three times to calculate the standard deviation (SD) and relative standard deviation (RSD) of the Ct value, to assess the reproducibility of the experimental results.

### 2.12. Detection in Biological Samples Collected from CSFV-Vaccinated Pigs

During the sample collection process, samples were obtained from six pig farms, in Jilin City, China, where no swine fever epidemics had been reported. A total of six samples was collected, which including 35 lymph samples, 28 meat samples, 40 throat swabs, 40 whole blood samples, and 50 fecal samples from 70 pigs that had been vaccinated within the past two weeks. To verify the accuracy of the test, the 193 samples were selected and tested using a commercial real-time qPCR kit for CSFV (Beijing Shiji Yuanheng Animal Disease Control Technology Co., Ltd., Beijing, China).

## 3. Results

### 3.1. Optimization of Dual TaqMan-MGB qPCR for CSFV and HCLV

The reaction conditions were studied by optimizing the primer and probe concentrations and the RT-qPCR program. Table 2 indicates that when the concentrations of the primers were 0.2 μmol/L and 0.4 μmol/L, the Ct value was higher and the repeatability was poor, and when the primer concentration was 0.6 μmol/L, the Ct value was the lowest. Thus, 0.6 μmol/L of the primer was used in this test. Considering the amplification efficiency, Ct value, and repeatability, the optimal concentration of the probes was determined at 0.1 μmol/L. The difference in the detection results among different annealing temperatures were small, and the annealing temperature was selected as 60 °C. Therefore, the final detection reaction system was set as follows: primer concentration 0.6 μmol/L, probe concentration (CSFV-P and HCLV-P) 0.1 μmol/L, and optimal annealing temperature 60 °C.

The optimized dual TaqMan-MGB qPCR reaction system was as follows: 15 µL of 2× Hifair^®^V C58P2 MP Buffer, 1.2 µL of Hifair^®^V C58P2 Enzyme Mix, 1.8 µL of the CSFV-F/CSFV-R (each at 10 µmol/L), 0.3 µL of the probe (CSFV-P and HCLV-P) (each at 10 µmol/L), 5 µL of template, and 4.6 µL of RNase Free H_2_O. The reaction program was as follows: 50 °C for 5 min; 95 °C for 10 s; 95 °C for 1 s, and 60 °C for 20 s, 45 cycles.

### 3.2. Preparation of the Standard Curve

**Figure 2:**
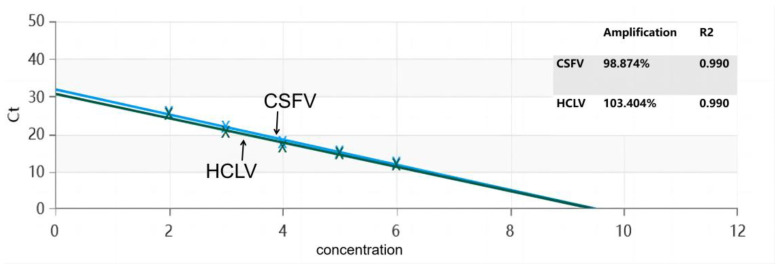
Standard curve for the dual RT-qPCR.

To establish a standard curve for the test, 10-fold serial dilutions of the CSFV-*NS3*-Plasmid and HCLV-*NS3*-Plasmid were used as DNA templates and subjected to TaqMan-MGB RT-qPCR. The detection results showed that this real-time PCR technique has a good linear relationship for the detection of CSFV-*NS3*-Plasmid, with an amplification efficiency of 98.874% and a correlation coefficient R^2^ = 0.99. For HCLV-*NS3*-Plasmid, the detection also had a good linear relationship, with an amplification efficiency of 103.404% and a correlation coefficient R^2^ = 0.99 (Figure 2).

### 3.3. Analytic Specificity

**Figure 3:**
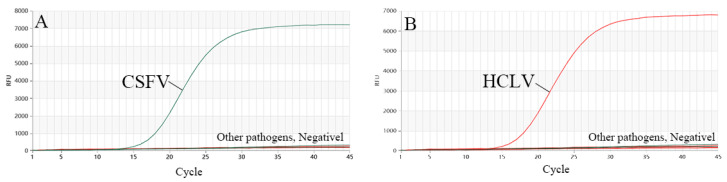
Specificity of dual RT-qPCR. (**A**) Detection result for CSFV, (**B**) detection result for HCLV.

The DNA samples prepared as described in Section 2.3 were used to evaluate the specificity of the test. Three repeated detections of dual TaqMan-MGB RT-qPCR showed that there were only specific amplification curves for CSFV, and no amplification curves were observed for HCLV and the other DNA samples or the negative control. In addition, dual TaqMan-MGB RT-qPCR also showed that there were only specific amplification curves for HCLV, and no amplification curves were observed for CSFV and the other DNA samples or the negative control, indicating that the dual TaqMan-MGB RT-qPCR method established in this study had good specificity (Figure 3).

### 3.4. Analytic Sensitivity

**Figure 4:**
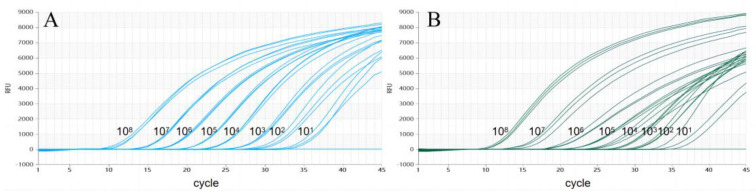
The detection limit of dual RT-qPCR. (**A**) Detection result for CSFV-NS3-Plasmid. (**B**) Detection result for HCLV-NS3-Plasmid).

To assess the analytic sensitivity of the test, 10-fold serial dilutions of the plasmid mixture were used for RT-qPCR (Figure 4). This showed that the analytic sensitivity of dual TaqMan-MGB RT-qPCR for *NS3* genes was 1.67 × 10^1^ copies/μL.

**Figure 5:**
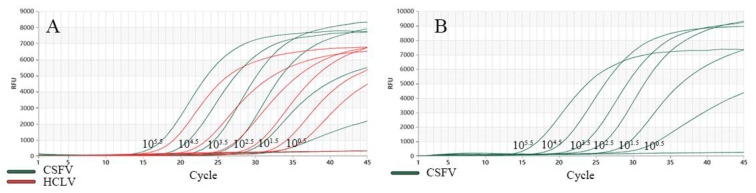
Detection limit testing. (**A**) The detection limit of dual RT-qPCR for CSFV and HCLV. The green amplification curves represent the detection result for CSFV, the orange amplification curves represent the detection result for HCLV (**B**) The detection limit of commercial qPCR kit for HCLV.

In addition, the analytic sensitivity of the dual TaqMan-MGB RT-qPCR method was also evaluated by testing the virus cultures. Figure 5 illustrates that a positive result (Ct value ≤ 35) was obtained for CSFV at a dilution degree of 10^0.5^, with good repeatability and stability. However, at a dilution of 10^−0.5^, no typical amplification curve was observed. This method demonstrated a detection sensitivity of 3.16 TCID_50_/μL for CSFV virus. Meanwhile, for HCLV, a positive result (Ct value ≤ 35) was obtained at a dilution degree of 10^0.5^, and no typical amplification curve was observed when the dilution degree was 10^−0.5^, indicating that the detection sensitivity of the test was 3.16 TCID_50_/μL for HCLV virus. Additionally, the detection limit of the commercial qPCR kit was also up to 3.16 TCID_50_/μL for HCLV virus.

### 3.5. Inclusiveness of the Test

**Figure 6:**
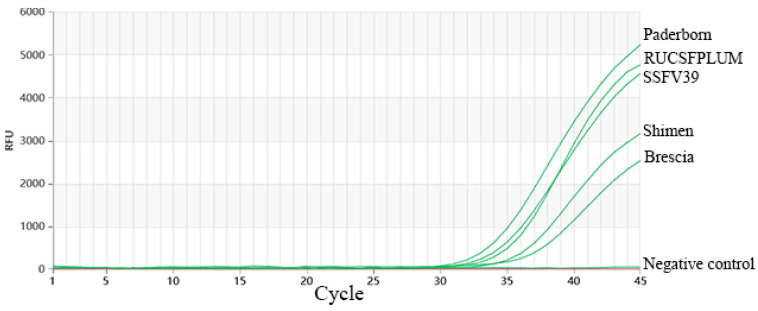
Inclusiveness of the test. The green amplification curves represent the amplification results of the pathogens, the horizontal straight lines represent the negative controls (ddH_2_O).

In this test, three repeated tests demonstrated that dual TaqMan-MGB RT-qPCR was able to identify five genotypes of strains, achieving a detection rate of 100%. The detection results are shown in Figure 6. This result showed that the newly developed technology has a good inclusiveness for the five classic epidemic strains.

### 3.6. Precision of the Test

The repeatability of the test was evaluated as described in Section 2.11. The RSD values were ≤2% (Table 3). Indicating that, the method developed in this study has a high detection precision.

For the reproducibility of the test, different instruments, reagent batches, laboratories, and operators were chosen for method reproducibility experiments. The results indicated that the SD values of the detection on three different concentrations of plasmid mixtures were less than 1.0, and the RSD values were less than 2.0%, which demonstrates that there was a good reproducibility of the method.

### 3.7. Detection in Biological Samples Collected from CSFV-Vaccinated Pigs

A total of 193 samples were collected and tested using this method. The results showed that the positive rate for CSFV detection was 16.1% (lymph samples, 2.0%; meat, 1.0%; pharyngeal swabs, 3.6%; whole blood, 3.6%; feces, 5.6%), and the positive rate for HCLV detection was 2.9% (lymph samples, 0%; meat, 1%; pharyngeal swabs, 0%; whole blood, 1.5%; feces, 0%). To verify the accuracy of the assay, 193 samples were tested using a commercial qPCR kit. The results showed that the positive rate for CSFV detection was 18.1% (lymph samples 2%, meat 1.5%, pharyngeal swabs 3.6%, whole blood 5.6%, feces 5.2%). The results showed that TaqMan-MGB dual RT-qPCR is comparable to the commercial qPCR kit (Table 4). The samples detected as positive for HCLV in the dual TaqMan-MGB RT-qPCR assay that could not be detected by the commercial qPCR kit were confirmed as positive using DNA sequencing. These results indicated that dual TaqMan-MGB RT-qPCR could be used for the differential diagnosis of CSFV and HCLV in field samples.

## 4. Discussion

Establishing a rapid, accurate, and specific method for differentiating between wild-type and vaccine strains of CSF is crucial for the subsequent prevention and control of CSF. Currently, there have been numerous studies on CSFV detection methods utilizing qPCR, with a primary focus on developing qPCR assays for CSFV. Limited research exists on distinguishing between wild-type CSFV and vaccine strains [15,16]. In this study, based on the alignment results of representative gene sequences of CSFV and HCLV, the highly conserved gene *NS3* was selected as the target for gene primer and probe design. Within the primers, two TaqMan-MGB probes were designed based on the SNP sites of CSFV and HCLV, with FAM and VIC fluorescent dyes labeled at the 5′ ends and MGB labeled at the 3′ ends of the probes. After systematic optimization of the RT-qPCR conditions, a one-step dual TaqMan-MGB fluorescent quantitative RT-PCR method for CSFV and HCLV was established.

In this test, the specificity, sensitivity, and detection ability of the biological samples collected from CSFV-vaccinated pigs were evaluated. This study showed that the method is specific for the detection of CSFV and HCLV, and it yielded negative results for 19 other common pig pathogens. The detection limits for CSFV and HCLV were 1.67 × 10^1^ copies/μL, respectively. The detection of CSFV and HCLV in 193 biological samples collected from CSFV-vaccinated pigs showed that the positive detection rates of CSFV and HCLV in the samples were 16.1% and 2.9%, respectively. The samples positive for HCLV in the dual TaqMan-MGB RT-qPCR assay were also confirmed to be positive by DNA sequencing. Indicating that dual TaqMan-MGB RT-qPCR can be used for the differential diagnosis of CSFV and HCLV in field samples.

Despite recent advances, the newly developed assay is also dependent on traditional multi-step DNA extraction before PCR amplification processes, which restricts its usefulness for on-site pathogen detection. Future research will investigate direct amplification qPCR as a possible solution for on-site CSFV and HCLV detection. In summary, the one-step dual TaqMan-MGB RT-qPCR technology established in this study meets the needs for early and rapid detection of CSFV, and can provide an efficient and rapid detection method for the investigation and analysis of CSFV field strain infections and vaccine immune status, thereby laying the foundation for better prevention and control of the occurrence and spread of CSF.

## 5. Conclusions

This study established a new one-step dual TaqMan-MGB RT-qPCR detection technology for the simultaneous differentiation of wild-type CSFV and vaccine strains. This technology has high specificity, high sensitivity, and high precision and has been successfully used for the detection of CSFV on pig farms. This technology is a useful tool for distinguishing natural infections from wild-type CSFV in vaccinated pig herds.

## Figures and Tables

**Table 1:** Primer and probe sequences.

Primer/Probe Name	Sequences (5′-3′)
CSFV-F	5′-ATAGGGGAGATGAAGGAAGG-3′
CSFV-R	5′-ACCCGCAGGTTCTCTGA-3′
CSFV-P	5′-6-FAM-ATCTGTGGATCTTCCCCATGA-MGB-3′
HCLV-P	5′-VIC-ATCTGTGAATCTTTCCCATGA-MGB-3′

Note: The same pair of primer was used for CSFV and HCLV.

**Table 2:** Optimization results of dual real-time RT-PCR.

Optimization Project	----	Average Ct Values (10^7^/10^6^/10^5^)	R^2^
Annealing temperature (CSFV)	58 °C	17.77/20.65/24.08	R^2^ = 0.99747
	60 °C	16.86/20.39/23.41	R^2^ = 0.99798
	62 °C	16.73/21.86/23.07	R^2^ = 0.88697
Annealing temperature (HCLV)	58 °C	18.52/22.25/24.32	R^2^ = 0.97342
	60 °C	18.42/22.12/24.24	R^2^ = 0.97602
	62 °C	18.22/22.95/24.03	R^2^ = 0.88374
Primer concentration (CSFV)	100 nM	16.95/19.76/21.92	R^2^ = 0.99433
	200 nM	18.36/20.63/23.30	R^2^ = 0.99782
	300 nM	17.22/19.54/23.87	R^2^ = 0.97045
	400 nM	16.64/19.53/24.05	R^2^ = 0.98413
Primer concentration (HCLV)	100 nM	16.43/20.20/23.48	R^2^ = 0.99839
	200 nM	18.65/21.28/25.01	R^2^ = 0.99013
	300 nM	17.65/21.78/25.03	R^2^ = 0.99528
	400 nM	18.30/21.14/25.92	R^2^ = 0.97885
Probe concentration	200 nM	17.43/20.20/26.42	R^2^ = 0.95321
	400 nM	19.35/22.82/24.37	R^2^ = 0.95351
	600 nM	16.31/21.00/24.18	R^2^ = 0.98788

**Table 3:** Detection precision for dual real-time RT-PCR.

		Ct-Value (CSFV/HCLV)						Standard Deviation	Relative Standard Deviation
		Gentier 96R			StepOne Plus				
Dates	Concentration	Lot No.20240608			Lot No.20240610				
06/11	10^3^ copies/µL	24.5/27.0	24.1/26.9	24.7/26.9	25.1/27.1	25.0/27.3	25.1/27.4	0.40/0.21	1.61%/0.77%
06/12		25.2/27.4	25.0/27.1	25.3/27.4	24.9/27.2	25.1/27.4	25.3/27.1	0.16/0.15	0.65%/0.55%
06/13		25.5/27.7	24.9/27.3	25.1/27.3	24.7/27.6	24.9/27.5	25.2/27.7	0.28/0.18	1.12%/0.67%
06/14		24.9/27.2	25.2/26.8	25.1/27.5	24.7/27.4	25.4/27.6	25.3/26.8	0.26/0.35	1.03%/1.28%
06/15		25.5/27.3	25.6/27.1	25.1/26.9	26.4/27.8	24.9/27.6	25.4/27.9	0.52/0.40	1.45%/2.03%
06/11	10^1^ copies/µL	32.5/34.4	32.4/34.3	32.4/34.4	32.2/34.6	32.4/33.9	32.8/34.1	0.20/0.25	0.60%/0.72%
06/12		32.6/34.4	32.8/34.6	32.2/34.1	32.6/34.3	32.9/33.8	32.4/34.1	0.25/0.278	0.78%/0.81%
06/13		32.2/34.4	32.1/34.3	32.9/34.5	32.6/33.9	32.7/34.3	32.6/34.4	0.30/0.20	0.94%/0.61%
06/14		31.9/34.4	32.6/34.2	31.9/34.1	31.9/34.8	32.3/34.5	32.4/34.6	0.31/0.26	0.96%/0.75%
06/15		32.5/34.4	32.8/34.1	32.4/34.6	32.8/34.9	33.3/35.1	33.0/34.8	0.33/0.36	1.00%/1.04%

**Table 4:** Comparison of dual real-time PCR and commercial kits.

Categories	Dual Real-Time PCR		Commercial Kit
	CSFV	HCLV	CSFV
Lymph node	4	0	4
Meat	2	2	3
Pharyngeal swab	7	0	7
Whole blood	7	3	11
Feces	11	0	10

## Data Availability

All data are included within this article and Supplementary Materials.

## Supplementary Materials

The following supporting information can be downloaded at: https://www.mdpi.com/article/10.3390/vetsci11070289/s1, Table S1. Vaccine details.
