# Supplementary material for: Rapid Differential Detection of Wild-Type Classical Swine Fever Virus and Hog Cholera Lapinized Virus Vaccines by TaqMan MGB-Based Dual One-Step Real-Time RT-PCR
Source: Vet Sci. 2024 Jun 28;11(7):289. doi: 10.3390/vetsci11070289 (PMC11281418; doi:10.3390/vetsci11070289)
Supplement: Supplementary file 1 [file vetsci-11-00289-s001.zip › vetsci-3009486-supplementary.pdf]

Table S1. Vaccine details

Name	Specifications	Production batch number	Source
High-pathogenicity Porcine Reproduction and Respiratory Syndrome vaccine, live (Strain JXA1-R)	10 copies/bottle	2304002	Zhongmu Industry Co., Ltd
Porcine Reproduction and Respiratory Syndrome vaccine, live (Strain R9)	10 copies/bottle	20230301	RuiPu Biopharmaceutical Co., Ltd
Porcine Reproduction and Respiratory Syndrome vaccine, live (Strain CH-1R)	10 copies/bottle	202303	Chengdu Shi Ji Biopharmaceutical Co., Ltd
Porcine Reproduction and Respiratory Syndrome vaccine, inactivated (Strain CH-1a)	20 mL/bottle	20230909	Wuhan Keda Biotechnology Co., Ltd
Swine Epidemic Encephalitis Vaccine, live (Strain SA14-14-2)	10 copies/bottle	20220721	Wuhan Keda Biotechnology Co., Ltd
Porcine Parvovirus vaccine, inactivated(Strain WH-1)	20 mL/bottle	20220817	Wuhan Keda Biotechnology Co., Ltd
Swine influenza virus H1N1 subtype, inactivated vaccine (TJ strain)	20 mL/bottle	20220502	Wuhan Keda Biotechnology Co., Ltd
Porcine pseudorabies vaccine, live vaccine (HB-98 strain)	10 copies/bottle	2212022	Zhongmu Industry Co., Ltd
Classical Swine Fever Live Vaccine (Cell-Derived)(CVCC AV1412 Strain)	10 copies/bottle	2201011	Zhongmu Industry Co., Ltd
Inactivated porcine circovirus disease type 2 vaccine (SH strain).	20 mL/bottle	072110006	PLeiro Biotechnology Engineering Co., Ltd
Porcine transmissible gastroenteritis, porcine epidemic diarrhea, porcine rotavirus (GP5 type) live vaccine (flower virus strain + CV777 strain + NX strain)	1 copies/bottle	2022020	Harbin Weike Biotechnology Co., Ltd
Foot and mouth disease type O and A bivalent 3B protein epitope deletion vaccine, inactivated (O/rV-1 strain + A/rV-2 strain)	20mL/bottle	0AA220618	Inner Mongolia Biwei Antai Biotechnology Co., Ltd
Haemophilus parasuis quadrivalent propolis inactivated vaccine (type 4 SD02 strain + type 5 HN02 strain + type 12 GZ01 strain + type 13 JX03 strain)	20 mL/bottle	202209003	Shandong Huahong Biotechnology Engineering Co., Ltd
Staphylococcus suis propolis inactivated vaccine (Staphylococcus suis group C BHZZ-L1 strain + Staphylococcus suis type 2 BHZZ-L4 strain)	20 mL/bottle	202209009	Shandong Huahong Biotechnology Engineering Co., Ltd
Trivalent inactivated vaccine for virulent colibacillosis in piglets (containing K88, K99, and 987P flagellar antigens)	10 mL/bottle	202209006	Shandong Huahong Biotechnology Engineering Co., Ltd
Porcine Mycoplasmal Pneumonia Inactivated Vaccine (Strain J)	20 mL/bottle	20220808	Shandong Huahong Biotechnology Engineering Co., Ltd
Triple live vaccine for swine fever, swine erysipelas and pasteurellosis (cell source + G4T10 strain + EO630 strain)	10 copies/bottle	2208037	Zhongmu Industry Co., Ltd

Inactivated vaccine against porcine atrophic rhinitis (Bordetella pertussis strain JB5)	20 mL/bottle	20220602	Wuhan Keda Biotechnology Co., Ltd
Porcine infectious pleuropneumonia trivalent inactivated vaccine (serotype 1 9901 strain, serotype 2 XT9904 strain, serotype 7 GZ9903 strain)	20 mL/bottle	20220706	Wuhan Keda Biotechnology Co., Ltd
